# Placental vascularization alterations in hypertensive disorders complicating pregnancy (HDCP) and small for gestational age with HDCP using three-dimensional power doppler in a prospective case control study

**DOI:** 10.1186/s12884-015-0666-1

**Published:** 2015-10-05

**Authors:** Ting Yuan, Ting Zhang, Zhen Han

**Affiliations:** Department of Obstetrics and Gynecology, First Affiliated Hospital of Xi’an Jiaotong University College of Medicine, Shaanxi, China

**Keywords:** Hypertensive disorders complicating pregnancy (HDCP), Placenta, Small for gestational age (SGA), Three-dimensional power Doppler (3DPD)

## Abstract

**Background:**

Hypertensive disorders complicating pregnancy (HDCP) continues to be a leading cause of maternal and neonatal mortality and morbidity. The clinical value of placental three-dimensional power Doppler (3DPD) in assessing HDCP requires further confirmation. The research was developed to assess changes of placental vascularity in HDCP using 3DPD and to investigate the placental vascularity in small for gestational age (SGA) compared with not-SGA in patients with HDCP.

**Methods:**

There were 126 normotensive and 128 hypertensive pregnant women included in this prospective case–control study from March 2011 to March 2013. Pregnant women underwent 3DPD. Vascularization index (VI), flow index (FI) and vascularization flow index (VFI) were obtained. The placental 3DPD indices, umbilical artery systolic and diastolic ratio (S/D) and pregnancy outcomes were compared between the groups.

**Results:**

The placental VI and VFI were significantly lower in hypertensive women compared with normotensive women (*P* < 0.001 and *P* = 0.014, respectively), and these parameters were significantly reduced in severe preeclampsia (*P* < 0.001 and *P* = 0.003, respectively). A weak correlation was found between VI and umbilical artery S/D in HDCP group (*r* = -0.277, *P* = 0.001). In HDCP population, neonates who were postnatally diagnosed with SGA had lower VI (*P* = 0.041) and higher S/D (*P* < 0.001).

**Discussion:**

The placental vascularity indices decreased in hypertensive women and the reduction inplacental perfusion was consistent with the severity of the hypertensive disorder. The associations betweenplacental vascularization and umbilical artery impedance may be valuable for further researches and arerequired confirmation. The significant differences in the 3DPD placental vascularization between SGA andnot-SGA in hypertensive pregnancy population may show some clinical importance that we could use tobetter assess or predict the progression and adverse outcomes in the future. Although 3DPD quantificationhas been widely used in multiple publications, we have to acknowledge its limitations.

**Conclusions:**

The intraplacental vascularization was poor in HDCP, and especially in severe preeclampsia. Neonates with SGA had poor placental vascularization and higher umbilical artery S/D. Further studies should focus on the clinical assessment of placental 3DPD as well as a combination of placental 3DPD and other fetal Doppler indices to better predict the development and outcomes of preeclampsia.

## Background

Hypertensive disorders complicating pregnancy (HDCP) continues to be a leading cause of maternal and neonatal mortality and morbidity [1]. The prevalence of HDCP is approximately 5.22 % of all pregnancies in China and 8–10 % worldwide [2, 3]. In recent years, important advances have been made in our understanding of the pathogenesis and pathophysiology of preeclampsia, but most of the factors that contribute to the disease remain unclear. Multiple tests and combination tests have been developed as screening methods for preeclampsia, including maternal serum biomarkers and Doppler parameters. To date, there is no effective screening test for HDCP [4–6].

HDCP is currently considered as a chronic placental disease. Placental dysfunction and hypo-perfusion play an important role in HDCP pathophysiology and are considered to be responsible for pathologic pregnancy outcomes, such as fetal growth restriction and perinatal loss [7–9]. Three-dimensional power Doppler (3DPD) has been a focal point of recent placental research; it is superior to 2D Doppler in several ways, including its ability to detect the secondary and tertiary stem vessels in the placenta and intraplacental vessel characteristics, such as the vessel density, branching, caliber changes and tortuosity, which can be shown using 3DPD [8, 10, 11]. Quantitative 3DPD analysis has been used to assess placental perfusion and vascularization indices, which potentially reflect both utero-placental and feto-placental blood perfusion [10].

Assessment of placental perfusion using 3DPD seems to be more helpful in understanding HDCP pathophysiology. However, the clinical value of placental 3DPD in assessing HDCP requires confirmation. The aim of our study was to compare changes in placental vascularity between HDCP and normotensive subjects using 3DPD, and to assess whether placental vascularity changes presenting in small for gestational age (SGA) compared with not-SGA in HDCP.

## Patients and methods

### Enrolment

This was a prospective case–control study that was conducted at the First Affiliated Hospital of Xi’an Jiaotong University, ShaanXi, China, from March 2011 to March 2013. Women with HDCP were enrolled, and normotensive pregnant women were used as controls. HDCP and normotensive pregnant subjects were individually matched by maternal age ± 2 years and gestational week at examination ±2 weeks.

The hypertensive group was further stratified into the following subgroups: (1) gestational hypertension (hypertension for the first time during pregnancy without proteinuria); (2) severe preeclampsia (diastolic BP ≥110 mmHg, systolic BP ≥160 mmHg; proteinuria from none to positive; elevated serum creatinine and transaminase levels; obvious fetal growth restriction (FGR); multi-organ disturbances, such as headache, visual disturbance, upper abdominal pain, oliguria, convulsion, thrombocytopenia and pulmonary edema); (3) nonsevere preeclampsia (diastolic BP <110 mmHg, systolic BP <160 mmHg; proteinuria from none to positive; no multi-organ disturbances or only minimal serum transaminase elevation). Nonsevere preeclampsia includes “mild” and “moderate” hypertension, which is not specifically defined. Overt proteinuria may not be a necessary element to characterize preeclampsia syndrome [12]. Hypertension was defined as systolic blood pressure (SBP) ≥140 mmHg and/or diastolic blood pressure (DBP) ≥90 mmHg on at least two occasions at a 6-hour interval.

All women with fetal chromosomal abnormalities, fetal malformations, placental anomalies (bipartite placenta or velamentous insertions), umbilical anomalies, complications associated with pregnancy (including diabetes mellitus, intrahepatic cholestasis, thrombophilia and thrombocytopenia), systemic vascular or autoimmune disorders, multiple pregnancies, or rupture of amniotic membrane as well as women receiving vasoactive drugs or those in active labor were excluded from the study. Control participants were healthy and normotensive pregnant women.

The study was approved by the ethics and research committee of the First Affiliated Hospital of Xi’an Jiaotong University. The purpose and procedures of the study were explained to all enrolled participants and written informed consent was obtained from each participant.

### 3DPD assessment

A single operator (Ting Yuan) performed all ultrasound scans using a Voluson E8 (GE Medical Systems, Milwaukee, WI, USA) ultrasound system. In addition to routine ultrasound, the placental 3DPD was performed in all women.

Placental vascularization was assessed using 3DPD. Because placental blood perfusion varied from region to region, we standardized the process by choosing five different sampling sites, according to Guiot [13]. The five spherical sampling sites in each placenta were as follows: one central, two peripheral at opposite sides and one between the central site and each peripheral site.

**Fig. 1:**
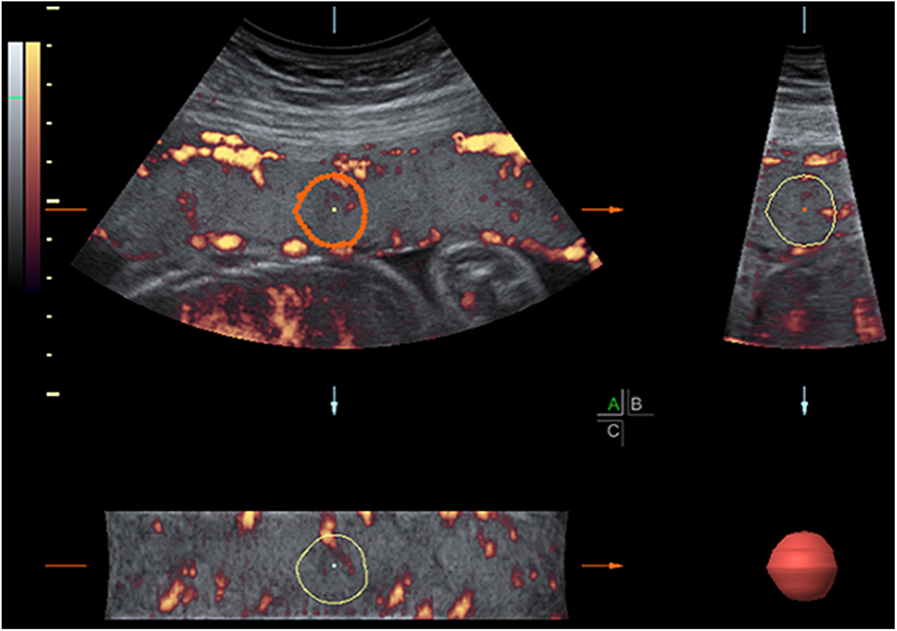
Assessment of the placental 3DPD at 25 weeks gestation in normal pregnancy

The peripheral sampling spheres were always positioned to maintain a minimum distance of approximately 4 mm between the insonated region and both the chorionic and basal plates. Maximal sensitivity was ensured using the following settings: pulse repetition frequency (PRF), 0.6 kHz; wall motion filter (WMF), low 1; frequency, mid; dynamic, 3; balance, 4150; smooth, 4/5; ensemble, 11; line density, 8; power Doppler map, 4; artefact suppression, off; power Doppler line filter, off; and quality, high. The placenta was investigated using a constant volume and the same 25-degree sector angle throughout. The spheres were kept away from the spiral arteries and they were placed near the basal and/or chorionic plate (Fig. 1). The 3D Glass Body render mode was used, in which the colour and the grey value information are processed and combined to give a 3D image. The vascularization indices were obtained using the Virtual Organ Computer-aided Analysis (VOCAL) program. The manual mode was set, and the five spherical regions of interest in each placenta were manually encircled by rotating 30 degrees six times, respectively. The sphere volume did not include vessels from the basal and chorionic plates, and it was kept constant. A histogram automatically showed the vascularization indices. The three-dimensional volume was formed using basic units called voxels. The voxel contains all information about the grey scale and colour, according to an intensity scale that ranges from 0 to 100. The vascularization indices are as follows: vascularization index (VI) refers to the colour voxel-to-total voxel ratio, indicating the number of vessels that could be detected within the placental volume; flow index (FI) refers to the weighted colour voxel divided by the total colour voxel ratio and provides an amplitude value for the colour signal, which indirectly estimates the placental blood flow during a 3D sweep; vascularization flow index (VFI) refers to the weighted colour voxel-to-total voxel ratio, which indicates a combination of vascularity and blood flow. The three index values were obtained from each sampling measurement, and the average value of the three indices from five measurements was provided for the final statistical analysis.

The systolic and diastolic ratio (S/D) of the umbilical artery Doppler measurement was used for the final analysis. Other records were reviewed to evaluate the pregnancy outcomes, including the delivery mode, delivery week, neonatal birth weight, Apgar scores and the diagnosis of small for gestational age (SGA). SGA was confirmed at birth by a newborn weight <2 standard deviations (SDs) below the mean for gestational age [14]. The gestational age was calculated using the last menstruation period (LMP) or confirmed based on the results of the first trimester ultrasound.

### Statistical analysis

Data gathered from the study subjects were verified and double-entered into a data management system. Variables are presented as the mean ± SD or median (range) and percentages, as appropriate. Statistical analysis was performed using the Pearson, *χ*^2^, Fisher exact, Student’s *t*, Mann–Whitney U, analysis of variance, Kruskall-Wallis and Steel Dwass tests, as appropriate. The following variables were compared between the groups: maternal age, gestational week at examination, parity, SBP, DBP, placental vascularity (VI, FI and VFI), umbilical artery S/D, delivery mode, delivery week, Apgar scores and SGA diagnosis. Correlation analysis was performed using Spearman rank analysis between placental vascularity and umbilical artery S/D. All reported P values were 2-tailed, and values <0.05 were considered statistically significant. Statistical analysis was performed using SPSS version 16.0 (IBM, Armonk, NY, USA) and GraphPad Prism version 5.0 (GraphPad, La Jolla, CA).

## Results

A total of 264 pregnant women were initially enrolled, but 2 were diagnosed with intrahepatic cholestasis, 2 were diagnosed with gestational diabetes and 6 were lost to follow-up. As a result, 126 normotensive women and 128 hypertensive pregnant women were included in the final analysis. In the hypertensive group, there were 38 patients with gestational hypertension, 40 patients with mild preeclampsia and 50 patients with severe preeclampsia.

Table 1 shows the characteristics of the normotensive and hypertensive pregnant women. There were similar maternal ages, gestational weeks at examination and parity in both groups. The SBP and DBP were significantly higher in the hypertensive group than in normotensive group. The placental VI and VFI were significantly lower in the hypertensive group than in the normotensive women (*P* < 0.001 and *P* = 0.014, respectively). The umbilical artery S/D increased significantly in the hypertensive group compared with the normotensive group (*P* = 0.001). Overall, hypertensive pregnant women (median delivery gestational age, 36.43 weeks) delivered earlier than pregnant women in the control group (median delivery gestational age, 39.57 weeks; *P* < 0.001). Additionally, the delivery mode was significantly different, with a higher frequency of caesarean section and induced labor with amniotic cavity injection in the hypertensive group compared with the normotensive group (*P* < 0.001). There were more neonates with 1-minute Apgar scores <7 and more neonates diagnosed with SGA in the hypertensive group compared with the controls (*P* < 0.001 and *P* = 0.004, respectively).

**Table 1:** Characteristics, placental vascularity and neonatal outcomes in the normotensive and hypertensive groups

Parameter^a^	Normotensive(*n* = 126)	Hypertensive(*n* = 128)	P
Age, yrs	29.14 ± 4.22	29.76 ± 4.82	0.341
Gestational age at examination,wks	35.00(22.57–42.00)	35.86(21.14–41.57)	0.320
Parity, n			0.381
Nulliparous	100	95	
Mutiparous	26	33	
SBP, mmHg	110(85–125)	150(130–230)	<0.001
DBP, mmHg	70(56–87)	100(90–158)	<0.001
VI	24.47(10.56–48.22)	18.49(10.49–49.55)	<0.001
FI	43.89(7.26–92.80)	41.50(1.02–97.70)	0.620
VFI	10.47(0.91–31.54)	7.55(0.11–44.20)	0.014
Um-S/D	2.23(1.44–3.55)	2.48(1.54–5.92)	0.001
Delivery mode, n			<0.001
vaginal	59	12	
cesarean	67	105	
Induced labor	0	11	
Gestational age at delivery,wks	39.57(34.14–42.00)	36.43(22.29–41.71)	<0.001
Postnatal SGA, n			<0.001
Yes	3	26	
No	123	91	
1-m-Apgar score, n			0.004
< 7	3	12	
≥ 7	123	105	

^a^ *SBP* systolic blood pressure, *DBP* diastolic blood pressure, *VI* vascularization index, *FI* flow index, *VFI* vascularization flow index, *Um-S/D* umbilical artery systolic and diastolic ratio, *SGA* small for gestational age

**Fig. 2:**
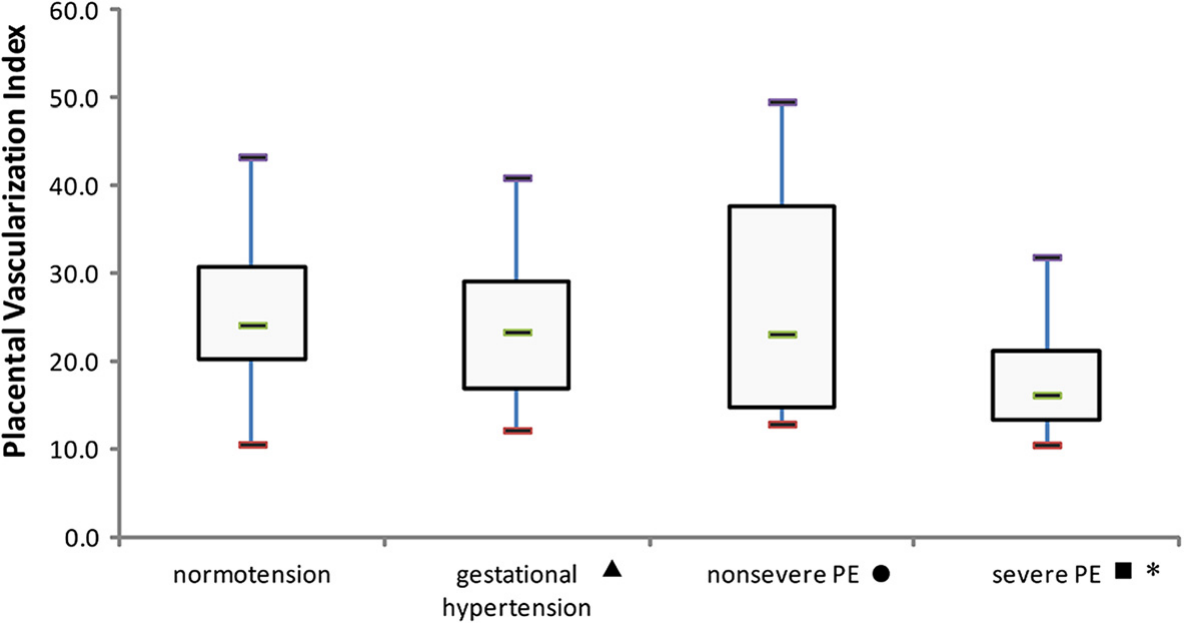
The placental VI in different population subgroups. The horizontal bar within each box corresponds to the median. The upper and lower bars of each box correspond to the first and third quartiles, respectively. The vertical whiskers outside each box extend to the smallest and largest observations within a 1.5 interquartile range (third-first quartiles). The symbols of ▲, ● and ■ represented the comparisons of parameters between normal group and gestational hypertension, normal group and nonsevere PE, normal group and severe PE. * represented a significant difference. PE = preeclampsia. For placental VI, P values of ▲, ● and ■ were 0.32, 0.61 and <0.001, respectively

**Fig. 3:**
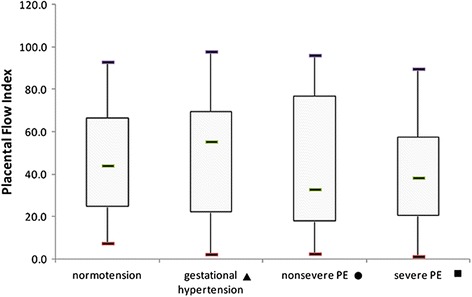
The placental FI in different population subgroups. The horizontal bar within each box corresponds to the median. The upper and lower bars of each box correspond to the first and third quartiles, respectively. The vertical whiskers outside each box extend to the smallest and largest observations within a 1.5 interquartile range (third-first quartiles). The symbols of ▲, ● and ■ represented the comparisons of parameters between normal group and gestational hypertension, normal group and nonsevere PE, normal group and severe PE. * represented a significant difference. PE = preeclampsia. For placental FI, *P* values of ▲, ● and ■ were 0.24, 0.81 and 0.24, respectively

**Fig. 4:**
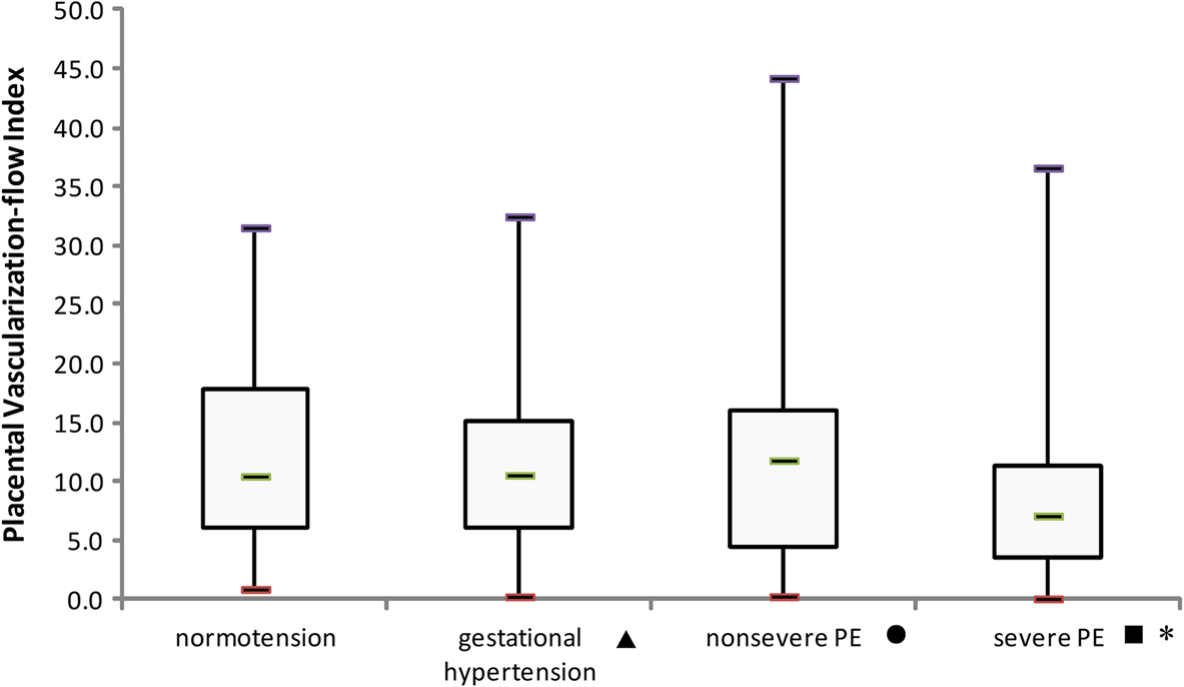
The placental VFI in different population subgroups. The horizontal bar within each box corresponds to the median. The upper and lower bars of each box correspond to the first and third quartiles, respectively. The vertical whiskers outside each box extend to the smallest and largest observations within a 1.5 interquartile range (third-first quartiles). The symbols of ▲, ● and ■ represented the comparisons of parameters between normal group and gestational hypertension, normal group and nonsevere PE, normal group and severe PE. * represented a significant difference. PE = preeclampsia. For placental VFI, P values of ▲, ● and ■ were 0.97, 0.70 and 0.001, respectively

**Fig. 5:**
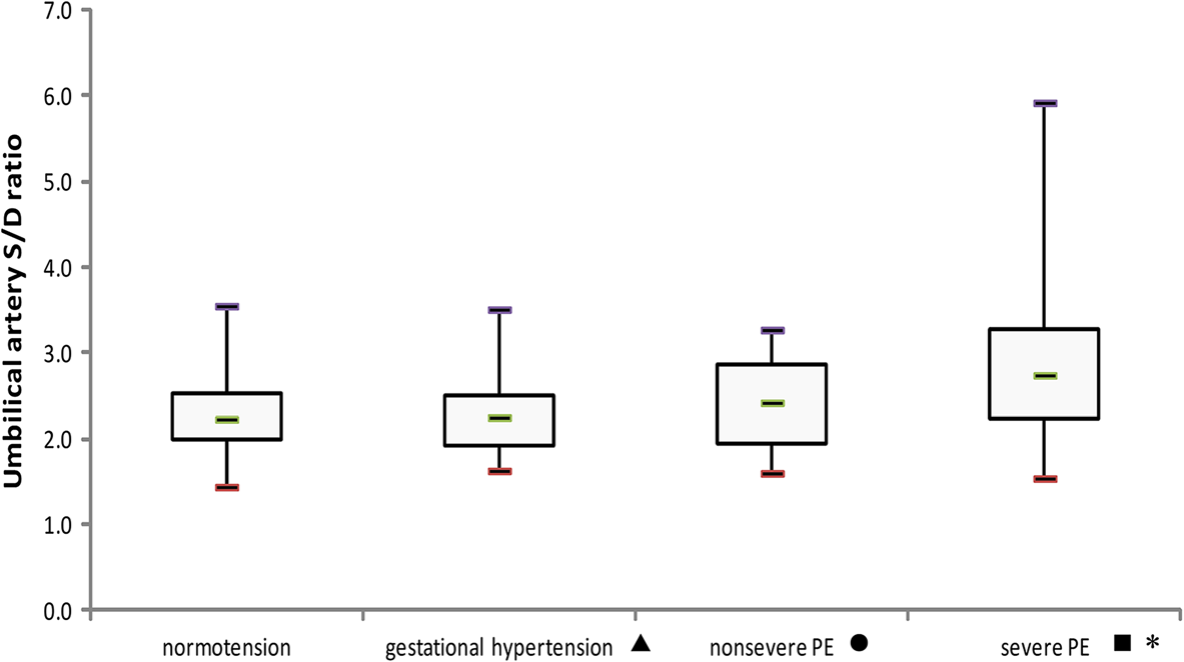
The umbilical artery S/D in different population subgroups. The horizontal bar within each box corresponds to the median. The upper and lower bars of each box correspond to the first and third quartiles, respectively. The vertical whiskers outside each box extend to the smallest and largest observations within a 1.5 interquartile range (third-first quartiles). The symbols of ▲, ● and ■ represented the comparisons of parameters between normal group and gestational hypertension, normal group and nonsevere PE, normal group and severe PE. * represented a significant difference. PE = preeclampsia. For umbilical S/D ratio, *P* values of ▲, ● and ■ were 0.69, 0.62 and <0.001, respectively

Table 2 shows the characteristics and results among normotensive patients and those with different types of hypertension. The demographic information was similar between the groups. Compared with normotensive subjects, patients with severe preeclampsia had a significant reduction in the placental VI (*P* < 0.001) and VFI (*P* = 0.001), as well as a significant increase in umbilical artery S/D (*P* < 0.001), but there was no difference with the other types of hypertension (Figs. 2, 3, 4 and 5). The delivery week, SGA diagnosis and 1-minute Apgar scores <7 were compared between groups, and patients with severe preeclampsia delivered the earliest and had the highest frequency of neonates with SGA as well as a 1-minute Apgar score <7.

**Table 2:** Characteristics, placental vascularity and neonatal outcomes in different subgroups

Parameter^a^	Normotensive (*n* = 126)	Gestational hypertension (*n* = 38)	Nonsevere PE^b^ (*n* = 40)	Severe PE^b^ (*n* = 50)	*P*
Age, yrs	29.14 ± 4.22	29.56 ± 5.21	28.96 ± 6.23	30.81 ± 5.56	0.371
Gestational age at examination, wks	35.00(22.57–42.00)	37.23(34.57–41.57)	36.50(34.00–39.25)	35.53(21.14–38.56)	0.070
Parity, n					0.131
Nulliparous	100	33	27	35	
Mutiparous	26	5	13	15	
SBP, mmHg	110(85–125)	133(130–145)	148(140–155)	180(160–230)	<0.001
DBP, mmHg	70(56–87)	95(90–98)	95(90–105)	130(115–158)	<0.001
VI	24.47(10.56–48.22)	23.38(12.17–40.94)	23.13(12.88–49.55)	16.22(10.49–43.06)	<0.001
FI	43.89(7.26–92.80)	55.21(2.06–97.70)	32.70(2.30–95.96)	38.16(1.02–89.54)	0.282
VFI	10.47(0.91–31.54)	10.56(0.28–32.48)	11.81(0.30–44.20)	7.09(0.11–36.62)	0.003
Um-S/D	2.23(1.44–3.55)	2.25(1.63–3.51)	2.42(1.60–3.27)	2.74(1.54–5.92)	<0.001
Delivery mode, n					<0.001
vaginal	59	9	3	0	
cesarean	67	29	37	39	
Induced labor	0	0	0	11	
Gestational age at delivery, wks	39.57(34.14–42.00)	38.86(33.86–41.71)	37.71(30.14–40.28)	34.79(22.29–41.00)	<0.001
Postnatal SGA, n					<0.001
Yes	3	6	7	13	
No	123	32	33	26	
1-m-Apgar score, n					<0.001
< 7	3	0	2	10	
≥ 7	123	38	38	29	

^a^ *SBP* systolic blood pressure, *DBP* diastolic blood pressure, *VI* vascularization index, *FI* flow index, *VFI* vascularization flow index, *Um-S/D* umbilical artery systolic and diastolic ratio, *SGA* small for gestational age

^b^ *PE* preeclampsia

Table 3 shows the correlation between placental vascularity and umbilical artery S/D. There were no correlations in the normotensive population. However, the correlation between VI and S/D was significant (*P* = 0.001) in the hypertensive population, but the correlation was relatively weak (*r* = –0.277).

**Table 3:** Correlation between placental vascularity and Um-S/D in normotensive and hypertensive population

Index^a^	Normotensive		Hypertensive	
	r	P	r	P
VI	0.132	0.248	−0.277	0.001
FI	0.014	0.904	−0.122	0.159
VFI	0.147	0.196	−0.148	0.088

^a^ *VI* vascularization index, *FI* flow index, *VFI* vascularization flow index

Table 4 shows the comparison of patient characteristics and placental vascularity based on a postnatal SGA diagnosis only in the hypertensive population (for 128 hypertensive pregnant women, 117 neonates were alive). Neonates that were diagnosed with SGA were born significantly earlier than those with normal birth weights. The placental VI and umbilical artery S/D were significantly different in hypertensive pregnancies, resulting in SGA neonates (*P* = 0.041 and *P* < 0.001, respectively).

**Table 4:** Characteristics and placental vascularity according to postnatal diagnosis of SGA in the hypertensive population

Parameter^a^	SGA^b^		P
	No(*n* = 91)	Yes(*n* = 26)	
Diagnosis, n			0.122
Gestational H	32	6	
Mild PE	33	7	
Severe PE	26	13	
Age, yrs	29.90 ± 4.88	30.20 ± 4.94	0.800
Gestational age at delivery, wks	37.57(31.71–41.71)	33.85(31.14–38.57)	<0.001
VI	19.15(10.49–47.47)	15.49(10.62–49.55)	0.041
FI	45.06(1.02–96.52)	38.97(3.94–97.70)	0.621
VFI	8.00(0.11–44.20)	8.93(0.48–32.39)	0.917
Um-S/D	2.36(1.54–5.92)	3.41(1.94–5.77)	<0.001

^a^gestational h = gestational hypertension, *PE* preeclampsia, *VI* vascularization index, *FI* flow index, *VFI* vascularization flow index, *Um-S/D* umbilical artery systolic and diastolic ratio

^b^ *SGA* small for gestational age

## Discussion

Quantitative 3DPD histogram assessments of placental vascularization and blood flow have become feasible because of recent advances [10, 15]. Merce LT et al. [15] and some other authors reported that there were positive correlations between gestational age and the placental vascularization indices, and more placental trees and placental blood perfusion are available as gestation progresses in normal pregnancies [16, 17]. However, de Paula CF et al. [18] demonstrated that all placental vascular indices estimated by 3DPD presented a constant distribution throughout gestation in spite of the increase in placental volume according to gestational age.

According to previous studies, the placental vascularity indices decreased in hypertensive women compared with normal pregnancies, which has been demonstrated by many authors [19–22]. In the current study, we also found that, compared with normotensive pregnancies, there was less intraplacental vascularization in hypertensive pregnant women, and intraplacental vascularization was the worst in women with severe preeclampsia. Intraplacental vascularization in gestational hypertension and nonsevere preeclampsia did not differ from that in the normotensive group. However, superimposed preeclampsia was also found to have the most significant reduction in placental perfusion when compared amongst hypertensive disorder subgroups [19]. These results reflected the reduction in placental perfusion, which was consistent with the severity of the hypertensive disorder. In preeclampsia, a disease that reduces the number of vessels in the placental territory, a lower VI value could be interpreted in the context of deficient trophoblast invasion of spiral arteries [20]. The reduction in VFI indicates that the number of placental trees and blood flow perfusion are decreased. The FI value could be lower, reflecting placental blood insufficiency, although this was not confirmed in the study. Damages to placental vessels may be relatively mild in gestational hypertension and nonsevere preeclampsia, or in compensatory stage, which presented with no significant alterations in the placental vascularity indices.

Both low impedance of placental blood flow and intraplacental hyperperfusion, which conveys oxygen and nutrients, are necessary for normal fetal development and survival during pregnancy. Placental dysfunction and hypoperfusion are part of the preeclampsia pathophysiology and they correlate with adverse fetal outcomes. Doppler measurement of the umbilical artery has been used for decades to assess the fetal condition. Elevated impedance of the umbilical artery and absent end diastolic velocity (AEDV) or reverse end diastolic velocity (REDV) denotes a higher risk of survival and adverse outcomes for the fetus [23, 24]. Therefore, we assessed the correlation between the placental vascularity and umbilical artery impedance. There was no correlation in normal pregnancies, but a correlation was present in the hypertensive population. A lower placental vascularization was associated with a higher umbilical artery impedance. Therefore, some fetal outcomes may be an indirect result of reduced placental blood flow. Nevertheless, this relatively weak correlation (*r* = −0.277) should be taken into account. Doppler measurement of the umbilical artery is usually influenced by various factors, such as fetal movement and the patient’s position; thus, further research is required to confirm the actual correlation.

Pomorski M et al. [25] evaluated the placental vascularity indices between normal pregnancies and FGR pregnancies, and the indices were all lower in FGR pregnancies. They further suggested that VI and VFI were the best parameters with the most favorable potential to identify FGR pregnancies. de Almeida PE et al. [19] studied 62 normotensive and 66 hypertensive pregnant women, and no placental vascularity differences were found in neonates who were SGA compared to normal-sized neonates in the entire population. We compared the placental vascularity and umbilical artery S/D based on the postnatal SGA diagnosis in only the hypertensive population. The neonates who were diagnosed with SGA had a significantly lower prenatal VI and a higher umbilical artery S/D. Other studies have shown that FGR and low birth weight were associated with higher umbilical artery resistance, low placental weight and placental hypermaturiy, higher frequency of placentas in the III-level and infarction in the group with a higher umbilical artery [26, 27]. These results were in accordance with our findings. There are dysplastic placental villi and fibrous sediments in the villi, increased areas of atherosclerosis, ischemia and infarctions, resulting in a decreased total cross sectional area of the placental vessels, placental hypo-infusion and hypoxia, increased resistance of fetal circulation, elevated umbilical artery S/D, FGR and low birth weight.

Hata T et al. [28] assessed placental perfusion at 18–22 weeks of gestation in a low-risk population to predict adverse obstetrical complications or outcomes, including FGR and HDCP. It is the first study to assess 3DPD placental vascularization at approximately 20 weeks of gestation in a low-risk population to evaluate whether reduced endovascular trophoblast invasion and uteroplacental artery remodeling occur in pregnant women who develop FGR or HDCP. However, there were no significant differences in the VI, FI, or VFI values between FGR and normal pregnancies or between HDCP and non-HDCP pregnancies. Possible explanations for this lack of difference were the low number of women who developed adverse outcomes and that most HDCP cases were classified as nonsevere type. In spite of this, some authors have reported different results [29, 30]. The VI and VFI between 11–14 weeks of gestation were significantly lower in the pregnant women who developed PE and VI, which may have potential usefulness for the detection of HDCP. Therefore, it remains unclear whether 3DPD can depict early impaired proliferation, migration, and invasion of trophoblasts into the maternal decidua and myometrium [28]. It is necessary to confirm the true predictive value of 3DPD for placental vascularization in a low-risk population. By contrast, the study design we performed was evaluate whether there were significant differences in the 3DPD placental vascularization between SGA and not-SGA in the hypertensive pregnancy population instead of the low-risk population. The findings in Tables 3 and 4 showed the clinical importance of variables that could be used in combination to better assess or predict the progression of high-risk pregnancies and their adverse outcomes in the future. As a result, early precautions and effective interventions could be made to improve the prognosis.

With respect to the reproducibility of 3DPD ultrasound placental index measurements, there is significant variability for placental 3D power Doppler vascular index values among previous studies [31]. Merce LT et al. [32] were the first to reveal the validity of 3DPD, confirming the favourable reproducibility of 3DPD vascular indices in normal pregnancies. Similarly, Hata T et al. [33] and other researchers also provided intra- and inter-observer reproducibility applying 3DPD parameters in the placenta [34–37]. However, though multiple publications and journal submissions have used 3DPD, the reliability and reproducibility of 3DPD parameters are still questioned. Lai PK et al. [38] showed a poor reproducibility at even the most fundamental level in normal pregnancies. Guimarães Filho HA et al. [37] demonstrated that the FI was the highest reproducibility index in normal pregnancies between 26 and 35 weeks. Guiot C et al. [13] investigated normal and FGR pregnancies between 23-37 weeks and reported that the FI showed the least variability between the samples and was lower than controls only in the group with severe impairment of feto-placental blood perfusion. The results by Costa J et al. [22] were consistent with this. However, the study on 11–14 weeks of normal pregnancy by Tuuli MG et al. [39] revealed that VI and VFI were the most reliable indices, while FI showed significant bias towards underestimation in placental sonobiopsy research. It is concluded that further investigations on the reproducibility of placental perfusion and quantification using VOCAL are still required before 3DPD is applied as a clinical tool to quantify placental blood perfusion in normal and complicated pregnancies.

This study had some limitations. While the method of estimating placental vascularization was standardized and we considered the vascularization variability throughout the placenta, the long duration required for the measurement seemed to be a disadvantage.

Although 3DPD quantification has been widely used in recent studies and publications in the field of obstetrics and gynecology, we acknowledge that PD quantification also has several methodological limitations for assessing vascularization [40, 41]. These limitations include the high dependence on gain settings, lack of standardized machine settings [42–45], differences in attenuation [46], and issues with reproducibility [47–49], which are capable of altering the VI, FI and VFI quantification. A meaningful interpatient comparison of vascularity cannot be made without standardization. These issues limit the clinical applicability of 3DPD imaging. One alternative that could compensate for the effects of machine settings, attenuation, organ position and other confounding factors is fractional moving blood volume (FMBV), which can provide a more robust estimation of vascularity [50–54]. However, this method was not performed in the current study, and more obstetrics investigations that use FMBV should be performed in the future. Additionally, the volumetric pulsatility index (vPI) assessing the volumetric vascular impedance with spatiotemporal image correlation (STIC) and VOCAL could overcome these deficiencies, representing an important step in the process of standardization [43]. These alternative methods will be performed in the future.

Additionally, Zalud I et al. [55] demonstrated that there was a significant difference in the uterine spiral vasculature volume between younger and older women and that obstetrical complications could be partially related to the decreased uterine spiral vasculature volume; moreover, parity influenced all placental 3DPD vascularity indices. In the current study, there were no significant differences in maternal age and parity between the groups. However, in studies on hypertensive pregnancies, it seems necessary to adjust or stratify these influencing factors to gain more accurate results in the future.

## Conclusion

The intraplacental vascularization was poor in HDCP, especially in patients with severe preeclampsia. Neonates who were diagnosed with SGA had poor placental vascularization and higher umbilical artery S/D. Further studies should focus on the clinical assessment of placental 3DPD as well as a combination of placental 3DPD and other Doppler indices that represent the fetus could better predict the development and outcomes in patients with preeclampsia.

## Abbreviations

3DPD: Three-dimensional power Doppler
FI: Flow index
HDCP: Hypertensive disorders complicating pregnancy
SGA: Small for gestational age
S/D: Systolic and diastolic ratio
UmA: Umbilical artery
VI: Vascularization index
VFI: Vascularization flow index
